# Supplementary material for: Protein Kinase A Controls the Melanization of Candida auris through the Alteration of Cell Wall Components
Source: Antioxidants (Basel). 2023 Aug 31;12(9):1702. doi: 10.3390/antiox12091702 (PMC10525270; doi:10.3390/antiox12091702)
Supplement: Supplementary file 1 [file antioxidants-12-01702-s001.zip › Table S1_Revised.pdf]

Table S1. *C. auris* strains and primers used in this study

Strains used in this study

Strain	Genotype	Parent	Reference
**B8441**	Wild-type		[1]
**YSBA4**	*bcy1*Δ::*NAT*	B8441	[2]
**YSBA13**	*tpk1*Δ::*NAT*	B8441	[2]
**YSBA17**	*tpk2*Δ::*NAT*	B8441	[2]
**YSBA21**	*cyr1*Δ::*NAT*	B8441	[2]
**YSBA24**	*tpk2*Δ::*NAT tpk1*Δ::*HYG*	YSBA17	[2]
**YSBA39**	*gpa2*Δ::*NAT*	B8441	[3]
**YSBA41**	*ras1*Δ::*NAT*	B8441	[3]
**YSBA46**	*gpr1*Δ::*NAT*	B8441	[3]
**YSBA55**	*pde1*Δ::*NAT*	B8441	[3]
**YSBA59**	*pde2*Δ::*NAT*	B8441	[3]
**YSBA61**	*cdc25*Δ::*NAT*	B8441	[3]
**YSBA63**	*ira2*Δ::*NAT*	B8441	[3]
**YSBA70**	*pde2*Δ::*NAT pde1*Δ::*HYG*	YSBA59	[3]
**YSBA173**	B9J08_000072Δ::*NAT*	B8441	This study
**YSBA174**	B9J08_000073Δ::*NAT*	B8441	This study
**YSBA175**	B9J08_000073Δ::*NAT*	B8441	This study
**YSBA177**	B9J08_000517Δ::*NAT*	B8441	This study
**YSBA178**	B9J08_002997Δ::*NAT*	B8441	This study
**YSBA179**	B9J08_002997Δ::*NAT*	B8441	This study
**YSBA216**	B9J08_000072Δ::*NAT* B9J08_000073Δ::*NEO*	YSBA173	This study
**YSBA217**	B9J08_000072Δ::*NAT* B9J08_000073Δ::*NEO*	YSBA173	This study
**YSBA219**	B9J08_000517Δ::*NAT* B9J08_002997Δ::*NEO*	YSBA177	This study
**YSBA220**	B9J08_000517Δ::*NAT* B9J08_002997Δ::*NEO*	YSBA177	This study
**YSBA221**	B9J08_000072Δ::*NAT*	B8441	This study
**YSBA222**	B9J08_000517Δ::*NAT*	B8441	This study

Primers used in this study

Name	Primer description	Sequence (5' to 3')
**B18295**	B9J08_000072 5'-flanking region primer L1	GGGGTCGCAACTTCAATCTA
**B18296**	B9J08_000072 5'-flanking region primer L2	TTCAGATCCACTAGTTCTAGATTGGTCTTGTACGGGTGTA
**B18297**	B9J08_000072 3'-flanking region primer R1	TTTCCAGAATTTCACTCTTAAACAAGGGGAGGTTGATTCG
**B18298**	B9J08_000072 3'-flanking region primer R2	ACGATTCCACGAGATCAAGG
**B18299**	B9J08_000072 Internal screening primer LP	TGGTCTCAACGGGCTTATTC
**B18300**	B9J08_000072 Internal screening primer RP	ACCATGGTCTGCTCCTCATC
**B18301**	B9J08_000072 5'-screening primer SO	AGTTTCGGCCACAGTGACTT
**B18302**	B9J08_000072 3'-screening primer SO2	CTGCACTGAGGCGTACCTTA
**B18303**	B9J08_000073 5'-flanking region primer L1	GAGACCCGTTGGAAGGTGTA
**B18304**	B9J08_000073 5'-flanking region primer L2	TTCAGATCCACTAGTTCTAGGATCTGGGTAGAGTTGGAAT
**B18305**	B9J08_000073 3'-flanking region primer R1	TTTCCAGAATTTCACTCTTAGAGTGGATTTTGGTGACAAA
**B18306**	B9J08_000073 3'-flanking region primer R2	ATGGGTGGGAATGCAATTTA
**B18307**	B9J08_000073 Internal screening primer LP	CTTCGGCACTGGTGACTTCT
**B18308**	B9J08_000073 Internal screening primer RP	CGCATTCTTCATCAGCATGT
**B18309**	B9J08_000073 5'-screening primer SO	GGTGGAACTGCTTCTTGAGC
**B18310**	B9J08_000073 3'-screening primer SO2	AACAGCGTGGAAGAAGGAGA
**B18384**	B9J08_000517 5'-flanking region primer L1	ATAAAGGCGCCATACACAGG
**B18385**	B9J08_000517 5'-flanking region primer L2	TTCAGATCCACTAGTTCTAGATGCACAGAAGGTCTGAATG
**B18386**	B9J08_000517 3'-flanking region primer R1	TTTCCAGAATTTCACTCTTATTGGGTTTGCTTATATCAAG
**B18387**	B9J08_000517 3'-flanking region primer R2	GTGGGAGGGGGTCATAAAAA
**B18388**	B9J08_000517 5'-screening primer SO	GCTGTCATGCATCGCTTTTA
**B18389**	B9J08_000517 3'-screening primer SO2	GGCAGGGTGAGGTATTCAAA
**B18391**	B9J08_000517 Internal screening primer LP	ATCCTGCTGACGTCTTGCTT
**B18392**	B9J08_000517 Internal screening primer RP	GGGATCTTGGGAGCAACATA
**B18393**	B9J08_002997 5'-flanking region primer L1	ATCCTGAGGCGTTCAAGAGA
**B18394**	B9J08_002997 5'-flanking region primer L2	TTCAGATCCACTAGTTCTAGGATACAGGCTTCTGATTTGA
**B18395**	B9J08_002997 3'-flanking region primer R1	TTTCCAGAATTTCACTCTTAGTTTATAAAAAATCTGTATA
**B18396**	B9J08_002997 3'-flanking region primer R2	AATGCCCATTCAAACAAAGC
**B18397**	B9J08_002997 5'-screening primer SO	GCCAATGGAGACGAGAAATC
**B18398**	B9J08_002997 3'-screening primer SO2	CGCAAACTCCTGGGCTATAC
**B18399**	B9J08_002997 Internal screening primer LP	GGGAATGTTTGTCAGCCAGT
**B18400**	B9J08_002997 Internal screening primer RP	CCACACACCAGGATTGTCAG
**B18437**	B9J08_000073 5'-flanking region primer L1 (NEO)	TTGAGCCGCCATTTACATTT
**B18438**	B9J08_000073 5'-flanking region primer L2 (NEO)	GTATTCTGGGCCTCCATGTCGATCTGGGTAGAGTTGGAAT
**B18439**	B9J08_000073 3'-flanking region primer R1 (NEO)	GAATGCTGGTCGCTATACTGGAGTGGATTTTGGTGACAAA
**B18440**	B9J08_000073 3'-flanking region primer R2 (NEO)	ATGGGTGGGAATGCAATTTA
**B18461**	B9J08_002997 5'-flanking region primer L1 (NEO)	TCAAGTCTCGGATGCAAATG
**B18462**	B9J08_002997 5'-flanking region primer L2 (NEO)	GTATTCTGGGCCTCCATGTCGATACAGGCTTCTGATTTGA
**B18463**	B9J08_002997 3'-flanking region primer R1 (NEO)	GAATGCTGGTCGCTATACTGGTTTATAAAAAATCTGTATA

**B18464**	B9J08_002997 3’-flanking region primer R2 (NEO)	TCTTTCGAAGCCTTTCCTGA
**B11103**	pV1025 forward-extended primer	CTAGAACTAGTGGATCTGAA
**B11104**	pV1025 reverse-extended primer	TAAGAGTGAAATTCTGGAAA
**B11105**	*NAT* split primer 1	CCATTGACTAAGGTTTTCCC
**B11106**	*NAT* split primer 2	TTCAGTAGCCAAACCCATC
**B11107**	pV1025 Diagnostic screening primer 1	TCAGTGGCAAATCCTAACC
**B11108**	pV1025 Diagnostic screening primer 2	AGAGAAAATACCCGTGACG
**B16376**	pTO149 forward-extended primer	GACATGGAGGCCCAGAATAC
**B16377**	pTO149 reverse-extended primer	CAGTATAGCGACCAGCATTC
**B16378**	*NEO* split primer 1	TGCTCCTGCCGAGAAAGTAT
**B16379**	*NEO* split primer 2	GCTCTTCGTCGAGATCATCC
**B16380**	pTO149 Diagnostic screening primer 1	ACATGGGGATGTATGGGCTA
**B16381**	pTO149 Diagnostic screening primer 2	TTTTCGCCTCGACATCATCT
**B11749**	*ACT1* qRT-PCR primer 1	TTGCTCCTGAAGAACACCCT
**B11750**	*ACT1* qRT-PCR primer 2	GCAGGAACGTTGAAGGTCTC
**B13289**	*CHS1* qRT-PCR primer 1	GCCTGAAAGTATCCCGGAGT
**B13290**	*CHS1* qRT-PCR primer 2	CCAAATCCTAGTCGCATGCC
**B13291**	*CHS2* qRT-PCR primer 1	CGGCAGAACAGTTTACGACC
**B13292**	*CHS2* qRT-PCR primer 2	GGGCTTCTGTCTCACCTCTT
**B13293**	*CHS3* qRT-PCR primer 1	GGAGAGAGAAGATGGGGCTC
**B13294**	*CHS3* qRT-PCR primer 2	GTGGTATTGTGGCAAACGGT
**B13295**	*CHS4* qRT-PCR primer 1	GGGTGAAGTTGTCGAATCGG
**B13296**	*CHS4* qRT-PCR primer 2	GGCACAGATGGAGAGCATTG
**B13297**	*CHS5* qRT-PCR primer 1	GTGGGTAAACTCGATGCGTC
**B13298**	*CHS5* qRT-PCR primer 2	TAACAATCGAGCCGGCTTTG
**B13299**	*CHS6* qRT-PCR primer 1	TGCACACATACATTGGCGAG
**B13300**	*CHS6* qRT-PCR primer 2	CTGGATCGTCTGCACACATG
**B13301**	*CHS7* qRT-PCR primer 1	GGTATTGTGGGTGCCTTGTG
**B13302**	*CHS7* qRT-PCR primer 2	ATACCCACATCGACCGAGTC
**B13303**	*CHS8* qRT-PCR primer 1	TCGAGTACAATGGTCCCGAG
**B13304**	*CHS8* qRT-PCR primer 2	CCACGCTCATTCTGTTAGGC
**B13305**	*CDA2* qRT-PCR primer 1	CAGCTCCAGTGGTCGATTTG
**B13306**	*CDA2* qRT-PCR primer 2	TTGCACGAACTCTGTTGTCG
**B17985**	B9J08_000072 qRT-PCR primer 1	TGGTCTCAACGGGCTTATTC
**B17986**	B9J08_000072 qRT-PCR primer 2	AAGGTCGTGGGTTTGTTGAC
**B17987**	B9J08_000073 qRT-PCR primer 1	AGTTCTTGGCCAGGATTGTG
**B17988**	B9J08_000073qRT-PCR primer 2	TACCTATGAAGGGCGCAAAC
**B17989**	B9J08_000517 qRT-PCR primer 1	TCCCTCCCGGAGAAACTTAT
**B17990**	B9J08_000517 qRT-PCR primer 2	CTCATGCCATCACCGTATTG
**B17991**	B9J08_002997 qRT-PCR primer 1	CGCAGAGCATATGGTTCAGA
**B17992**	B9J08_002997 qRT-PCR primer 2	TTCAATGGTGTGGCACTGTT

References

1. Munoz, J.F.; Gade, L.; Chow, N.A.; Loparev, V.N.; Juieng, P.; Berkow, E.L.; Farrer, R.A.; Litvintseva, A.P.; Cuomo, C.A. Genomic insights into multidrug-resistance, mating and virulence in *Candida auris* and related emerging species. *Nat Commun* **2018**, *9*, 5346, doi:10.1038/s41467-018-07779-6.
2. Kim, J.S.; Lee, K.T.; Lee, M.H.; Cheong, E.; Bahn, Y.S. Adenylyl cyclase and protein kinase a play redundant and distinct roles in growth, differentiation, antifungal drug resistance, and pathogenicity of *Candida auris*. *mBio* **2021**, *12*, e0272921, doi:10.1128/mBio.02729-21.
3. Kim JS, L.K., Bahn YS. Deciphering the regulatory mechanisms of the cAMP/PKA pathway and their roles in the pathogenicity of *Candida auris*. *Microbiology Spectrum* **2023**, doi:10.1128/spectrum.02152-23.
