# Supplementary figures and images for: Protein Kinase A Controls the Melanization of Candida auris through the Alteration of Cell Wall Components
Source: Antioxidants (Basel). 2023 Aug 31;12(9):1702. doi: 10.3390/antiox12091702 (PMC10525270; doi:10.3390/antiox12091702)

Figure S1 (Kim et al.)

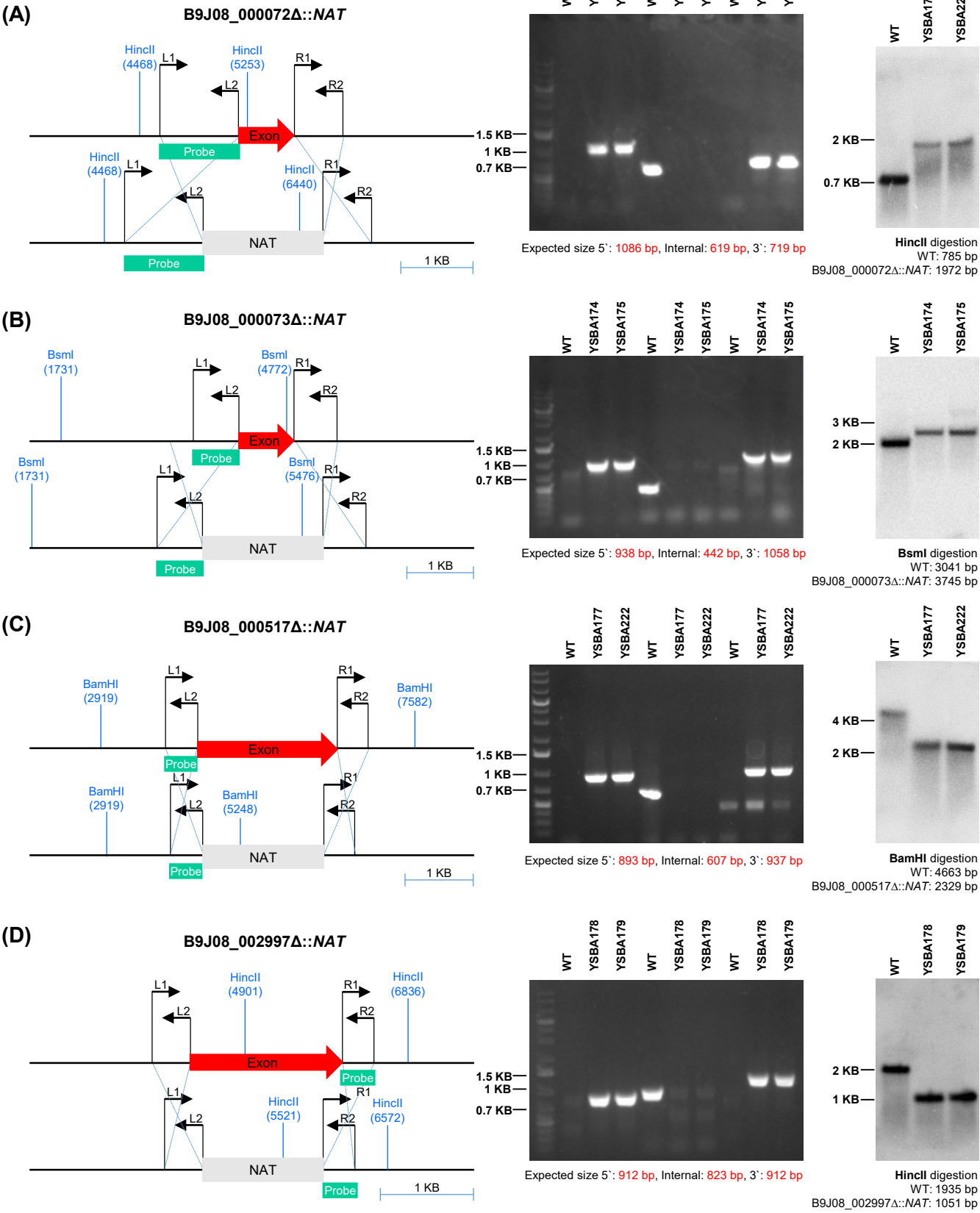

Figure S1 (Kim et al.)

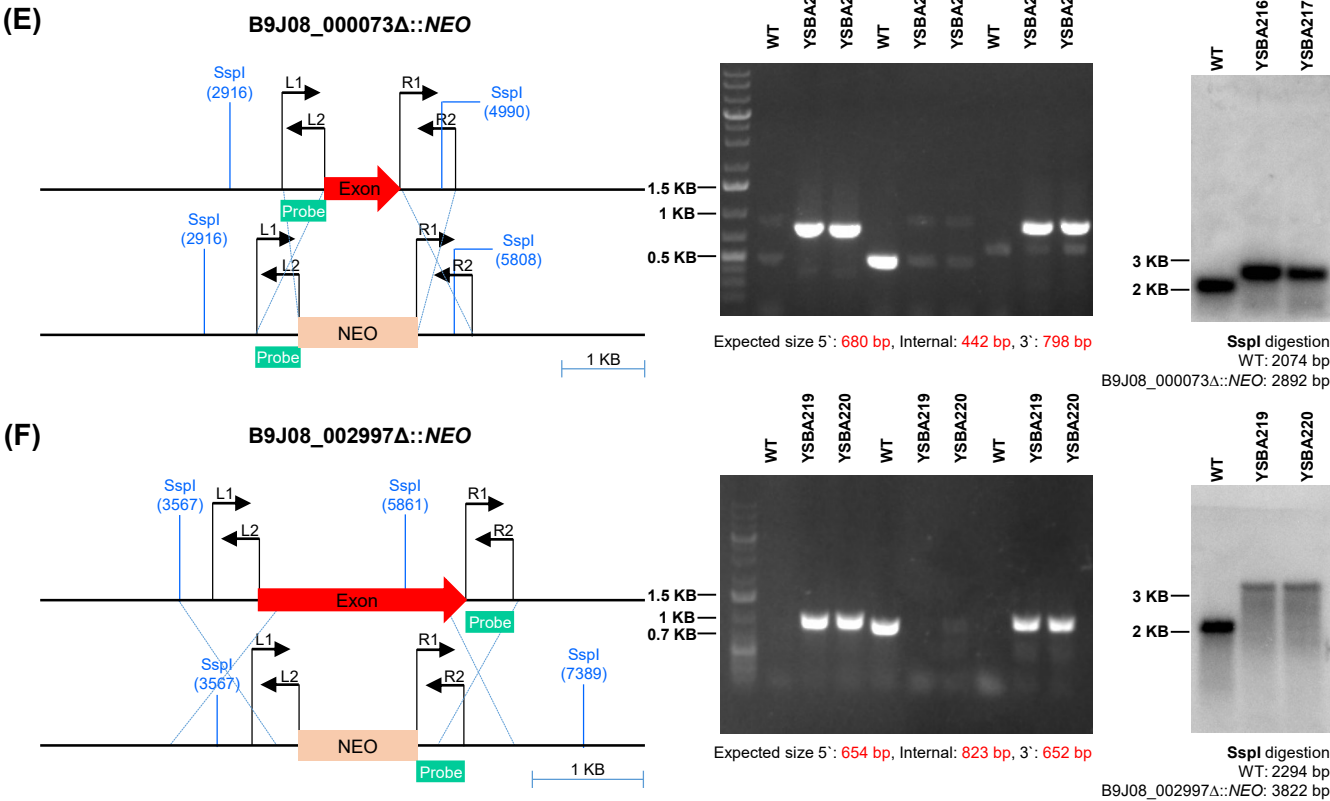

Supplement: Supplementary file 1 [file antioxidants-12-01702-s001.zip › Figure S1 (Kim et al).pdf]
